# Neoadjuvant administration of hydroxychloroquine in a phase 1 clinical trial induced plasma Par-4 levels and apoptosis in diverse tumors

**DOI:** 10.18632/genesandcancer.181

**Published:** 2018-05

**Authors:** Peng Wang, Ravshan Burikhanov, Rani Jayswal, Heidi L. Weiss, Susanne M. Arnold, John L. Villano, Vivek M. Rangnekar

**Affiliations:** ^1^ Department of Internal Medicine, University of Kentucky, Lexington, Kentucky, USA; ^2^ Markey Cancer Center, University of Kentucky, Lexington, Kentucky, USA; ^3^ Department of Radiation Medicine, University of Kentucky, Lexington, Kentucky, USA

**Keywords:** clinical trial, Par-4, tumor apoptosis, hydroxychloroquine

## Abstract

Chloroquine and hydroxychloroquine (HCQ) are robust inducers of the tumor suppressor Par-4 secretion from normal cells. Secreted Par-4 causes paracrine apoptosis of tumor cells and inhibits metastasis in mice. We report the clinical results with pharmacodynamic analyses of our Phase I trial using neoadjuvant administration of HCQ in patients with surgically removable early stage solid tumors. This was a single-institution trial of oral HCQ (200 or 400 mg twice daily) given for 14 days prior to planned surgery. Dose escalation was based on isotonic regression to model safety and biological effect based on plasma Par-4 analysis. Eight of the nine patients treated with HCQ showed elevation in plasma Par-4 levels over basal levels. No toxicities were observed with these dose regimens. The resected tumors from the eight HCQ-treated patients with elevated plasma Par-4 levels, but not the resected tumor from the patient who failed to induce plasma Par-4 levels, exhibited TUNEL-positivity indicative of apoptosis. Resected tumors from all nine HCQ-treated patients showed p62/sequestosome-1 induction indicative of autophagy-inhibition by HCQ. Our findings indicate that both dose levels of HCQ were well-tolerated and that Par-4 secretion but not induction of the autophagy-inhibition marker p62 correlated with apoptosis induction in patients' tumors.

## INTRODUCTION

Prostate apoptosis response-4 (Par-4, also called PAWR) is a tumor suppressor protein that is ubiquitously expressed in normal cells and tissues [1]. Par-4 induces apoptosis in diverse cancer cells but not in normal cells [1], yet it is often inactivated, down-regulated or mutated in several types of cancers [2]. Par-4 is located in various cellular compartments, including the cytoplasm, endoplasmic reticulum, and the nucleus, and both intra-and extracellular (i.e., secreted) Par-4 play a role in apoptosis induction by caspase-dependent mechanisms [1, 3, 4]. Moreover, Par-4 sensitizes cells to the action of diverse therapeutic agents [1]. Accordingly, loss of Par-4 in tumors contributes to recurrent tumors and a decrease in overall patient survival [5, 6]. Par-4 protein is secreted in cell culture-conditioned medium (CM) or systemically in mice by normal cells, and extracellular Par-4 binds to its receptor GRP78 on the cancer cell surface and induces FADD/caspase-8/caspase-3-dependent apoptosis [7]. By contrast, normal cells express low to undetectable levels of cell surface GRP78 and are resistant to apoptosis by extracellular Par-4 [7, 8].

Baseline levels of Par-4 secreted by normal cells are generally inadequate to cause massive apoptosis in cancer cells [8]. Our recent studies have identified small molecules such as chloroquine (CQ) and hydroxychloroquine (HCQ) that induce secretion of Par-4 from normal cells and cause apoptosis and inhibition of tumor metastasis by a Par-4-dependent mechanism [8]. CQ or its analog, HCQ, is an anti-malarial drug that acts as a robust inducer of Par-4 production from normal cells, via activation of p53 and Rab8b expression [8]. Importantly, CQ and HCQ inhibit the autophagic pathway in cells by blocking fusion of the autophagosome with the lysosome, yet they induce Par-4 via the classical secretory pathway that is sensitive to brefeldin-A, and that is independent of the autophagic pathway [8]. Context is essential in autophagy as it paradoxically can have both pro-death or pro-survival functions [9].

HCQ differs from CQ by the presence of a hydroxyl group at the N-ethyl substituent that is beta-hydroxylated, but has similar pharmacokinetics to CQ, with quick gastrointestinal absorption [10–11]. Importantly, HCQ is eliminated by the kidney with minimal side effects on short term treatments [12], making it an ideal choice for clinical trials. In clinical trials, CQ showed encouraging results in subsets of diverse cancers [13]. CQ induced cytotoxic effects in tumors by blocking autophagy, but in mouse pancreatic tumors containing oncogenic K-ras and lacking functional p53, loss of autophagy accelerated tumor progression [14]. CQ has been reported to display pleiotropic mechanisms of action that include inhibition of autophagy by blocking fusion of the autophagosome with the lysosome, lethal lysosomal destabilization, and normalization of tumor vasculature [15–17]. Although several clinical trials have been recently performed with HCQ [13], they tested HCQ in combination with standard-of-care anti-cancer therapy and none of them determined the relationship between pro-apoptotic Par-4 protein levels elevated in patients' plasma or serum and tumor response to the treatment.

We tested the hypothesis that as Par-4 induces apoptosis in diverse tumors, and as HCQ is expected to induce Par-4 secretion from normal cells and elevate plasma levels of Par-4 in a broad range of patients, HCQ may cause tumor cell apoptosis to inhibit the growth of tumors. Here, we report the results of a phase I clinical trial with two-week neoadjuvant oral administration of HCQ in patients with surgically removable early stage solid tumors. In addition to safety evaluation, we assessed biological response defined as induction of Par-4 levels from pre- to post-treatment plasma samples. Our studies indicate that HCQ inhibited autophagy, as judged by elevated p62 (also called sequestosome 1, SQSTM1) expression [18], in the tumors of all patients in the study. However, tumor cell apoptosis was detected only in patients exhibiting elevation of circulating levels of Par-4 protein in the plasma, implying that induction of Par-4 secretion but not autophagy-inhibition, correlated with tumor response to HCQ.

## RESULTS

### Patient characteristics

A total of nine patients with early stage solid malignancies were consented and enrolled in this study between December 2015 and November 2017. The median age of the subjects was 62 years (ranging from 54 to 78 years) with ECOG performance status of 0. Four patients had prostate adenocarcinoma, two patients had non-small cell lung cancer (NSCLC), and the other patients had diverse malignancies, including papillary thyroid carcinoma, squamous cell carcinoma of larynx, and carcinoid tumor of lung. Demographic and clinical characteristics of the patients are listed in Table 1.

**Table 1:** Patient characteristics (*N* = 9)

Characteristic	*N* (%)
**Sex**	
Male	8 (89)
Female	1 (11)
**Age (years)**	
Median	62
Range	54-78
**ECOG performance status**	
0	9 (100)
**Primary tumor site**	
Prostate adenocarcinoma	4 (44)
NSCLC, squamous cell type	2 (22)
Papillary thyroid carcinoma	1 (11)
Squamous cell carcinoma of larynx	1 (11)
Carcinoid tumor of lung	1 (11)

### Safety and dose escalation

All nine patients received 14 days of HCQ and were evaluable for toxicities. No HCQ-related dose limiting toxicities or serious adverse events (AEs) were noted. Table 2 shows safety and toxicity data for all patients enrolled in the trial. Patient 3, a 78-year-old gentleman with diagnosis of NSCLC at right lower lobe of lung, enrolled in dose level 1 in January 2016 and finished 14 days of HCQ at 200 mg daily without AEs observed. His surgical procedure was complicated with sepsis and adult respiratory distress syndrome with respiratory failure. He passed away in March 2016. These complications were deemed not related to HCQ.

**Table 2:** Summary of toxicities, DLT and Par-4 response

Dose Level^*^	Pt #	Type of Cancer	Adverse Event (Grade)	DLT	Par-4 (Fold) Response	
					______ ________	
					>2	>1.5 – <2
1	1	Papillary thyroid carcinoma	**None**	No	Yes	No
	2	Prostate adenocarcinoma	**None**	No	Yes	No
	3	NSCLC, squamous cell type	Diarrhea (Grade 1) Abdominal pain (Grade 3)	No	Yes	No
2	4	Squamous cell carcinoma of larynx	Floaters (Grade 1) Anorexia (Grade 1)	No	Yes	No
	5	Prostate adenocarcinoma	**None**	No	Yes	No
	6	Prostate adenocarcinoma	Blurred vision (Grade 1) Chest pain, cardiac (Grade 3) Thromboembolic event (Grade 2)	No	No	Yes
1	7	Prostate adenocarcinoma	**None**	No	No	Yes
	8	NSCLC, squamous cell type	**None**	No	No	No
	9	Carcinoid cancer of lung	Nausea (Grade 1) Urinary tract infection (Grade 2) Nausea (Grade 1)	No	Yes	No

^*^Dose Level 1 = 200 mg oral twice a day (*n* = 6); Dose Level 2 = 400 mg oral twice a day (*n* = 3)

**Figure 1:**
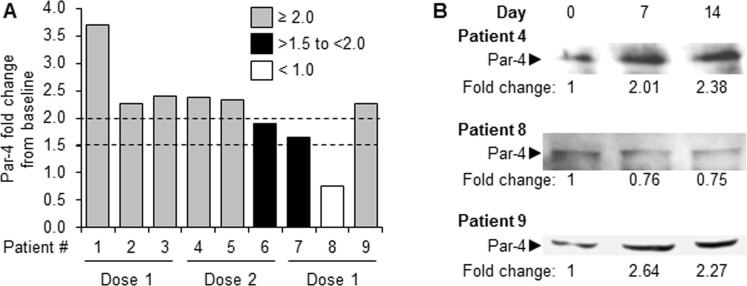
Induction of plasma Par-4 in cancer patients treated with HCQ **A.** Fold increase of Par-4 levels post-HCQ treatment. Plasma samples from the patients were collected pre-HCQ (Day 0), and Day 14 post HCQ treatment and analyzed by Western blot for Par-4 levels. Fold increase at Day 14 post-treatment relatively to pre-treatment (Day 0) levels is shown. **B.** Representative western blots for Patient 4, Patient 8, and Patient 9 are shown. Fold increase in Par-4 levels at Day 7 or Day 14 post-HCQ treatment relative to Day 0 pre-treatment levels is indicated.

Dose enrollment started with 3 patients at dose level 1 (200 mg twice daily). No dose limiting toxicity (DLT) was observed and all 3 patients exhibited a two-fold induction of Par-4 levels (Table 2 and Figure 1A). Toxicity and Par-4 response data were fitted into an isotonic regression model [19] for the next recommendation of 3 patients at dose level 2 (400 mg twice daily). No DLT was observed at dose level 2, but only 2 out of 3 patients achieved a two-fold induction in Par-4 levels. Likewise, using toxicity and Par-4 data from all patients enrolled thus far, the dose recommendation for the next cohort of 3 patients was dose level 1. No DLT was observed and 1 out of 3 patients achieved a two-fold increase in Par-4 in this next cohort of patients. Thus, no DLTs were observed for all nine patients enrolled in the study and Par-4 response was achieved in 67% of patients from both dose levels indicating the optimal biologic dose (lowest dose exhibiting a high biological response) was dose level 1 (200 mg twice daily).

### HCQ induced robust Par-4 secretion

Par-4 levels in the patients' plasma, before and after HCQ treatment, were quantified as described in Materials and Methods. Eight of the nine patients (except patient 8; dose level 1) demonstrated an increase (>1.5 fold) in plasma Par-4 levels on day 14 post-HCQ treatments compared to baseline pre-treatment levels (Figure 1A and 1B). Four out of six patients (number 1, 2, 3, 9) from dose level-1 and two out of three patients (number 4 and 5) from dose level 2 showed 2-fold or more elevation of plasma levels of Par-4 relative to pre-treatment baseline levels (Figure 1A). Two patients (number 6 and 7) showed >1.5 to <2-fold Par-4 elevation in plasma following HCQ treatment. Patient 8 (dose level 1) did not show any increase in plasma Par-4 levels following HCQ treatment (Figure 1A). Representative Par-4 western blots are shown for patient 4 and patient 9 who demonstrated >2-fold increase in plasma Par-4 levels with HCQ treatment, and for patient 8 who did not show Par-4 induction after HCQ treatment (Figure 1B).

### HCQ induced Par-4 caused paracrine tumor cell apoptosis

**Figure 2:**
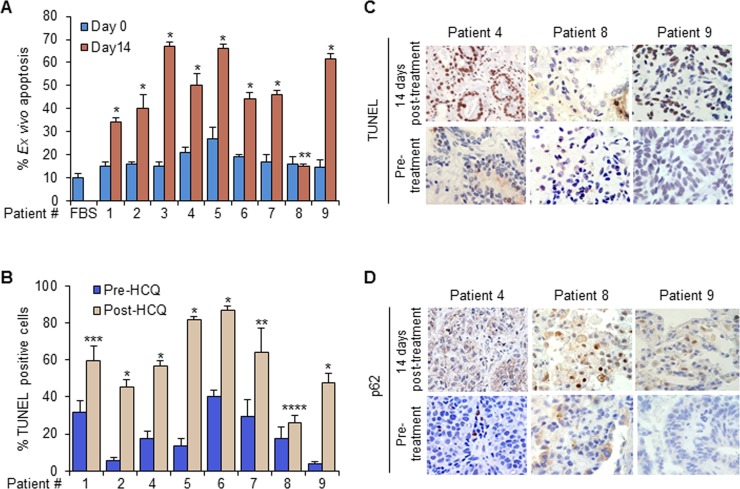
HCQ induced apoptosis and p62 levels in tumors **A.** Plasma from HCQ-treated patients caused ex vivo apoptosis in cancer cells. Aliquots of pre-treatment (Day 0) or post-HCQ treatment (Day 14) plasma or 10% FBS as control were added to H460 lung cancer cell line. After 24 hours, the cells were scored for apoptosis. Mean of three independent experiments + SD are shown. ^*^P < 0.0001, ^**^P=0.8955. **B.** HCQ induces apoptosis in patients' tumors. Diagnostic biopsies and paired resected tumor specimen were analyzed by TUNEL assay. TUNEL positive cells were scored relative to total number of cells and percentages are presented. Mean of three separate tumor sections + SD are shown. ^*^P< 0.0001, ^**^P = 0.0012, ^***^P = 0.0002, by the mixed linear model. For the TUNEL data from patient 8, ^****^P = 0.0343 by the mixed linear model or *P* = 0.077 by the paired t-test. **C.** Representative results of TUNEL assay. Diagnostic biopsy tissues (Pre-treatment) and paired resected tumor specimens (14-day Post-treatment) from the patients were subjected to TUNEL assays. Representative images of tumors from patients 4, 8, and 9 are shown. **D.** Representative results of p62 IHC staining. Diagnostic biopsy tissue (Pre-treatment) and paired resected tumor specimens (14-day Post-treatment) from the patients were subjected to p62 IHC assays. Representative images of tumors from patients 4, 8, and 9 are shown.

To determine the biological significance of HCQ induced Par-4 in plasma, aliquots of post-HCQ treatment plasma were added to H460 human cancer cells. All the post-HCQ treatment plasma samples, except that from patient 8, caused *ex vivo* apoptosis of the cancer cells (Figure 2A). Furthermore, TUNEL assays for *in vivo* apoptosis analysis were performed on the diagnostic biopsies and paired resected tumor specimens. All resected tumors from the eight patients who showed elevated expression of Par-4 in the plasma exhibited increase in TUNEL-positivity, relative to pre-treatment biopsy, indicative of apoptosis (Figure 2B). By contrast, the tumor resected from patient 8, who did not respond to HCQ by elevated secretion of Par-4, showed only marginal TUNEL-positivity difference relative to pre-treatment biopsy (Figure 2B). The pre-treatment biopsy specimen was not available for patient 3. Representative results of TUNEL assay on tumor tissues from patients 4, 8 and 9 are shown (Figure 2C).

### HCQ inhibited autophagy

The effect of HCQ on autophagy inhibition was analyzed by immunohistochemistry for p62, a biomarker for autophagosome-lysosome fusion inhibition. All

resected tumor specimens, including that from patient 8, were p62-positive, implying autophagy inhibition by HCQ and patient compliance of HCQ administration in all patients. Representative results are shown for p62 IHC staining from patients 4, 8, and 9 demonstrating positive IHC for p62 in resected tumor specimen after 14-day HCQ treatment relative to the corresponding pre-HCQ paired biopsies (Figure 2D).

## DISCUSSION

Our previous studies have indicated that HCQ induces secretion of Par-4 and that plasma samples collected following HCQ treatment of mice and patients exhibit *ex vivo* apoptotic activity that is neutralized by the Par-4 antibody. However, the relationship between elevated Par-4 secretion and apoptosis in patients' tumors was not known, providing a strong rationale for conducting this phase I trial. A total of 9 patients with early stage solid malignancies were enrolled in this trial and allocated to 200 mg twice daily or 400 mg twice daily cohorts. Our clinical results demonstrate significant induction of plasma Par-4 levels with both dose levels in cancer patients. No HCQ related toxicities were observed in these dose levels. After 14-day administration of HCQ, four out of six patients (67%) from dose level 1 and two out of three patients (67%) from dose level 2 showed a 2-fold or more elevation of plasma levels of Par-4 relative to pre-treatment baseline levels. Given these toxicity and Par-4 results, the optimal biological dose, defined as the lowest safe dose with a biological response was identified as 200 mg twice daily (dose level 1).

Based on these promising safety and biological results and other clinical trial priorities at our institution including a follow-up adjuvant study of HCQ, patient enrollment for this trial was terminated. Pharmacodynamics studies demonstrated *ex vivo* apoptosis of lung cancer cells with aliquots of post-HCQ treatment plasma from all patients who showed HCQ-induced plasma levels. By contrast, aliquots of plasma from patient 8, who did not show elevated plasma Par-4 levels with 14-day administration of HCQ, did not induce *ex vivo* apoptosis of the lung cancer cells. Importantly, resected tumors from all patients, except patient 8, exhibited remarkably increased TUNEL-positivity indicative of tumor cell apoptosis. On the other hand, all tumors, including that from patient 8, showed p62 induction indicative of inhibition of autophagy. These results indicated that HCQ inhibited autophagy in all patients' tumors but caused apoptosis only in the tumors of patients who showed elevated Par-4 secretion in their plasma in response to HCQ. Thus, elevated Par-4 secretion and not autophagy-inhibition by HCQ correlated with tumor cell apoptosis by HCQ. It is possible that apoptosis (TUNEL positivity) in the tumors post-HCQ treatment may be due to the combined effects of the increase in plasma Par-4 levels and autophagy-inhibition by HCQ.

It is particularly interesting that one patient (number 8) did not respond to HCQ by elevated secretion of Par-4. Our previous studies have indicated that induction of plasma Par-4 from normal cells is p53-dependent [8]. Mutation of *TP53* in normal cells is usually associated with Li-Fraumeni syndrome, an inherited autosomal dominant disorder that is manifested by a wide range of malignancies at an unusually early age [19]. However, patient 8 did not have clinical evidence of Li-Fraumeni syndrome, and further investigation on the underlying cause of the lack of Par-4 induction or tumor cell apoptotic response to HCQ in this patient is warranted.

In summary, this is the first clinical study indicative of HCQ as a robust inducer of Par-4 in plasma that correlated with tumor apoptosis in cancer patients. As the regimen of HCQ 200 mg twice daily for two weeks was safe, the results of this trial provided the justification for an adjuvant study (NCT03015324) that is now ongoing, to further illustrate the effects of HCQ on plasma Par-4 and potential clinical efficacy in preventing tumor relapse. Despite the sample size, the promising findings of this study have significant implications for expanding the clinical indications of HCQ, by conducting prospective, cancer-specific, adjuvant phase 2 clinical trials with long-term use of HCQ for prevention of tumor recurrence.

## MATERIALS AND METHODS

### Study design and objectives

This was a single-institution, phase I, open-label, dose finding trial of oral HCQ for 14 days prior to planned surgery. The study was reviewed and approved by the Institutional Review Board at University of Kentucky. The primary objective of the phase I study was to determine the effect of HCQ on plasma Par-4 protein levels in adults with resectable solid tumors and to compare them to pre-HCQ treatment plasma Par-4 levels. A secondary objective was to evaluate the toxicity profile of HCQ in this setting. Dose limited toxicity (DLT) was defined as a grade 3 or above toxicity with attribution (possibly, probably or definitely related) to the study medication that occurs during or within 30 days of the last dose of HCQ.

### Eligibility criteria

Patients above 18 years of age with early stage solid malignancies, according to the classification of the American Joint Committee on Cancer were eligible. All these malignancies were histologically confirmed and were planned for surgical resection without requirement for any neo-treatment per NCCN guideline. Other criteria included ECOG performance status ≤2, ability to ingest oral medications (crushing and administering via PEG tube was acceptable), normal hematological, adequate renal and liver function (transaminases ≤4 times the upper limits of the institutional normal), ability to understand and provide written informed consent. Exclusion criteria included pregnancy or breast feeding, metastatic cancer and/or cancer that was not amenable to surgery, significant malabsorption, on enzyme-inducing anti-epileptic medications.

### Treatment plan

Treatment was administered on an outpatient basis. Patients obtained HCQ by submitting prescriptions to our institute pharmacy and received HCQ every day for 14 days, starting at least 14 days before planned surgery and optimally ending one day prior to surgery. Tablets of HCQ were available in 200 mg strength. HCQ was administered in divided doses (twice a day) for doses above 200 mg/day to minimize nausea. The divided doses were taken in the morning and at night with meals. Subjects were required to keep a medication diary and to present this at the end of treatment. Patients were instructed that if they have emesis and regurgitate the medication within 30 minutes of taking it, the dose may be repeated once, but if vomiting occurred longer than 30 minutes after ingestion, the dose was not to be repeated. Subjects taking antacids, proton-pump inhibitors or H2-blockers were asked not to take HCQ within 4 hours of these medicines.

### Blood collection for analysis of plasma Par-4

Collection of specimens. Blood plasma samples were collected pre-dosing, and weekly during the preoperative period (+/− 1 day), as well as on or after surgery (+/− 1 day). Venous blood (4 ml) was withdrawn for Par-4 biomarker studies. Samples were withdrawn into sodium heparinized collection tubes.

Handling of specimens. After collection, blood and anti-coagulant were mixed by inverting the tube 8–10 times. Blood samples were placed on ice immediately and centrifuged within 30 min at 7200 g at 4°C for 2 min. Plasma was transferred into amber plastic tubes and stored on dry ice prior to storing at −80°C until analysis.

### Western blot analysis and quantification of Par-4 levels in plasma

Plasma was diluted 1:5 in Laemmli sample buffer, then boiled and 10 µl amounts were resolved by SDS-PAGE. After transfer of the resolved proteins to PVDF membrane, the blot was subjected to Western blot analysis with the Par-4 antibody (R334, SantaCruz Biotechnology, Inc.). Par-4 levels in plasma were normalized to albumin bands by subjecting parallel SDS-PAGE gels to Coomassie blue staining.

### *Ex vivo* apoptosis assay

Plasma samples from patients were added to the growth medium of lung cancer cells H460 (from ATCC, MD) at 10% final concentration and the cells were grown for 24 h. Apoptotic cells were identified by immunocytochemical analysis for active caspase-3, and apoptotic nuclei were revealed by 4, 6-diamidino-2-phenylindole (DAPI) staining. A total of three independent experiments were performed and approximately 500 cells were scored in each experiment for apoptosis under a fluorescent microscope.

### Tumor collection for TUNEL and autophagy assay

Diagnostic biopsies and paired resected tumor specimen were stored in formalin fixed, paraffin-embedded blocks for potential autophagy and apoptosis analysis. Each subject signed the consent form for tissue collection before being enrolled in the study. TUNEL assay was performed using Millipore ApopTag Peroxidase In Situ Apoptosis Detection Kit (Cat#S7100), and p62 IHC was performed using the p62 antibody from Cell Signaling (#88588) by Dana Napier at the Biospecimen and Tissue Procurement Shared Resource Facility of the Markey Cancer Center.

### Statistical methods

Given the established safety profile of HCQ, we were interested in confirming safety and assessing biological response based on Par-4 levels in plasma for this Phase I trial. An adaptive, nonparametric, isotonic regression model was employed in order to determine the biological effect of HCQ as well as to assess safety [20]. Biological effect was defined as a two-fold induction in Par-4 levels from pre- to post-treatment plasma samples. The target safety and Par-4 biological response rates were 30% and 70%, respectively. Three dose levels were proposed with patient enrollment starting at dose level 1. Three patients per cohort were enrolled with a potential maximum sample size of 18 patients. Dose escalation and de-escalation proceeded based on determining an admissible set of safe doses and within this set, a nonparametric isotonic estimate was utilized to determine dose level recommendations and the optimal biologic dose [20]. An optimal biologic dose for this study is defined as the lowest administered dose exhibiting the highest biologic effect while safe.

Descriptive statistics were calculated to summarize safety data, clinical and biological variables. The proportion of patients exhibiting a two-fold Par-4 increase as well as changes from pre to post-treatment levels are summarized descriptively. All *ex-vivo* experiments were performed in triplicate to verify the reproducibility of the findings. The results show a mean of at least three replicates +/− SD. Statistical analyses were carried out by the Markey Cancer Center Biostatistics and Bioinformatics Shared Resource Facility. *P* values were calculated using repeated measures linear mixed models for *ex-vivo* Par-4 and apoptosis markers.
